# Application of the Biospeckle Method for Monitoring Bull’s Eye Rot Development and Quality Changes of Apples Subjected to Various Storage Methods—Preliminary Studies

**DOI:** 10.3390/s120303215

**Published:** 2012-03-07

**Authors:** Anna Adamiak, Artur Zdunek, Andrzej Kurenda, Krzysztof Rutkowski

**Affiliations:** 1 Institute of Agrophysics, Polish Academy of Sciences, Doswiadczalna 4, 20-290 Lublin 27, Poland; E-Mails: a.zdunek@ipan.lublin.pl (A.Z.); a.kurenda@ipan.lublin.pl (A.K.); 2 Fruit Storage and Processing Department, Division of Pomology, Research Institute of Horticulture, Pomologiczna 18, 96-100 Skierniewice, Poland; E-Mail: Krzysztof.Rutkowski@inhort.pl

**Keywords:** biospeckle, apple, bull’s eye rot, shelf life

## Abstract

In this study, the biospeckle technique was evaluated for monitoring of apple bull’s eye rot development and product quality in general, during storage under various conditions and during subsequent shelf life. This non-destructive optical method is based on the analysis of laser light variations scattered from the sample. Apples of the cultivars ‘Pinova’ and ‘Topaz’, susceptible to bull’s eye rot, were used in two independent experiments. In the first, apples were non-destructively monitored for five months during cold storage. After that time, 34% of ‘Pinova’ and 21% of ‘Topaz’ apples displayed visible surface lesions. The increase of biospeckle activity was observed during the development of fungal disease. In the second experiment various storage conditions were used and apples were tested during their shelf life by non-destructive and destructive methods. This study showed that biospeckle activity decreased during shelf life, irrespective of storage conditions.

## 1. Introduction

Bull’s eye rot, caused by species of *Neofabraea,* better known as *Pezicula*, is one of the most frequent and damaging fungal diseases affecting stored apples. It starts in the orchard, but its symptoms appear only several months after harvest (generally after 3–4 months in cold storage). Late maturing apple cultivars like ‘Pinova’ and ‘Topaz’ are particularly susceptible to the disease, with an incidence that can exceed 15–30% after 120 days of cold storage [1–5]. Early detection of infection is important for ensuring microbiological quality and safety of food commodities. Actually, non-destructive techniques has been applied in the evaluation and monitoring of biological properties [6].

The biospeckle technique is a relatively new, non-invasive method to analyze the vitality of biomaterials. It is based on the optical phenomenon occurring during illumination of samples by coherent light. The scattered rays interfere with each other and form random, granular patterns consisting of dark and bright spots. If the illuminated sample does not show any biological activity, the images obtained are invariant. In the case of biological samples, however, the intensity distribution evolves and fluctuates in time [7,8]. This was first observed by Abramson at the Stockholm Royal Institute of Technology [9], who noticed that ‘when an apple is illuminated with laser light the speckles move!’. According to Braga *et al.* [10] processes such as cytoplasmic streaming, organelle movement, cell growth and division during fruit maturation and biochemical reactions are responsible for certain biospeckle activity. Brownian motion should also be taken into account [8]. Nevertheless knowledge about the biospeckle phenomenon in relation to fruits and vegetables is still limited, and there is a lack of consistent biological interpretations of the phenomenon.

Applications of the biospeckle technique in the agricultural area include monitoring of quality and ripening of fruits and vegetables, analysis of seeds or assessment of motility parameters [7,8,11–21]. In all cases the biospeckle activity changed with the state of investigated sample. Analysis of temporal variation of the speckles was used to evaluate the presence of fungi in beans [22]. Due to the fact that the laser light can penetrate apple tissue to a depth of 7–10 mm [7] it is possible to obtain information about biospeckle activity from tissue localized under the skin. This means that, there would be a chance to detect and monitor the development of different defects before their symptoms become visible on the fruit surface.

The aim of this work was to monitor bull’s eye rot development by means of the biospeckle technique. Up to now, examples of biospeckle application for estimation of fruits quality have been shown [12,14,16–19], however the influence of storage methods on biospeckle dynamics in post-storage shelf life was not tested yet. The second aim was to analyze changes of biospeckle activity and quality attributes of apples during shelf life after storage under normal and controlled atmosphere conditions, including 1-metylocyclopropene treatment.

## 2. Materials and Methods

Apples (*Malus domestica* Borkh.) of the cultivars ‘Pinova’ and ‘Topaz’ were obtained from the Research Institute of Horticulture in Skierniewice, Poland. These cultivars were chosen due to their known susceptibility to bull’s eye root. About 480 apples of each cultivar were harvested in October 2010 at their optimal harvest windows. For two experiments, the fruit were divided: (1) into a batch of 100 apples for non-destructive monitoring of bull’s eye rot development, and (2) into a batch of about 380 apples for destructive measurements during shelf life after storage under various conditions.

In the first experiment, fruits were stored under a normal atmosphere (NA) at 2 °C for about 140 d. One hundred apples were tested non-destructively at eight dates with 20 d intervals. About eight apples were removed from NA, immediately tested at room conditions and then placed back to NA. In total, about 15 min were required for all measurements.

In the second experiment apples were stored under normal atmosphere at 2 °C (1NA, 2NA, 3NA, which mean respectively 1, 2 and 3 months of storage), and under controlled conditions (1 °C, 2% CO_2_, 2% O_2_) for eight months (4CA). Additionally, one batch (5MCP) was treated by 1-metylocyclopropene (625 ppb, SmartFresh 03 VP, Rohm and Haas, Philadelphia, PA, USA) and then stored for eight months under CA atmosphere. In each of the five storage variants 75 apples were tested in shelf life simulation. Biospeckle activity and quality attributes (firmness, soluble solids content, dry matter content) were evaluated at 1, 3, 5, 7 and 10th day of shelf life.

### 2.1. Biospeckle Measurement

**Figure 1.**
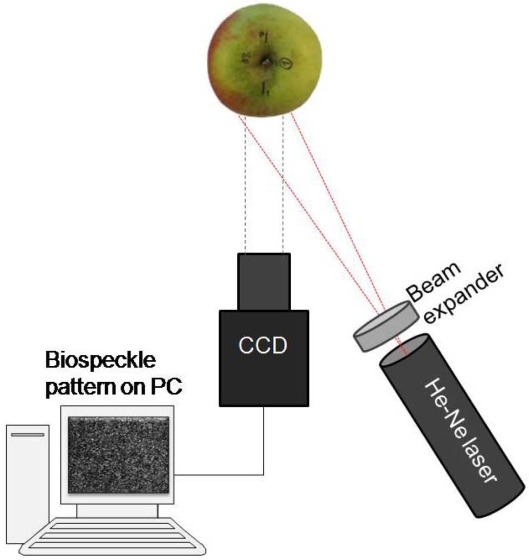
Scheme of the biospeckle setup.

The device for biospeckle measurement was similar to that previously used by Zdunek *et al.* [16–19]. Its schematic illustration is presented in Figure 1. In this study, two similar systems, equipped with differed laser for illuminating the sample were used.

In the first experiment, and in the case of 1NA, 2NA and 3NA series from the second experiment, a He-Ne laser (1 mW, λ = 632.8 nm, LLR 811, Optel, Opole, Poland) was used. The laser beam was expanded by a microscope objective (10/0.24, 160/-, PZO, Poland).

For the 4CA and 5MCP experiments, a diode laser (8 mW, λ = 635 nm, LQC635-08C, supplied with Laser Diode Control Unit, Newport, Irvine, CA, USA) was used. The laser beam was expanded by a beam expander 20× (Edmund Optics GmbH, Karlsruhe, Germany).

In both systems, a CCD camera (Monochrome FireWire Astronomy Camera DMK 21AF04.AS, The Imaging Source Europe GmbH, Bremen, Germany) with a 25 mm TV lens 1:14 and 20 mm extension ring (Pentax Corporation, Tokyo, Japan) was used as detector of scattered light. The distance between camera and apple was about 100 mm, and that between laser and apple 180 mm. The illumination angle was Θ ≈ 30°. Biospeckle movies lasting 4 s were recorded in uncompressed AVI film form (8 bits, RGB24 codec) at a 15 fps rate. The image exposure time of the CCD camera was 1/250, gain and brightness were set experimentally to avoid pixel overexposure on the image histogram. The image resolution was 320 × 240 pixels which corresponded to observation area of about 3 × 2 mm. These settings ensured avoidance of apple curvature. The parameters of image acquisition were kept unchanged during the experiment.

Biospeckle activity was evaluated using the correlation coefficient C^kτ^, where k is a frame number and τ is a lag time (1/15 s) [18,19]. C^kτ^ was calculated as the correlation coefficient of data matrix, consisting of intensities of pixels, of the first frame with the data matrixes of the following frames from the analyzed biospeckle movies. In this study, C^4^ was analyzed only as the correlation coefficient between the first frame and the frame at kτ = 4 s. The time of 4 s was chosen to obtain a reasonable decrease of C^kτ^ which was down to 0.2 for some apples and in the case of lasers and optics used. Then, a BA = 1-C^4^ value was determined as the biospeckle activity parameter for a certain sample. Higher biospeckle activity corresponds to higher 1-C^4^ value. Correlation coefficient C was calculated using “*corrcoef*” function in Matlab® R2010a software (MathWorks, Natick, MA, USA). Each apple was tested at six points localized on the fruit equator in the experiment 1, or at two opposite sites in experiment 2. As a result, mean values of BA were calculated.

### 2.2. Firmness Measurements

Firmness of the apples was measured in a non-destructive way with an acoustic impulse response technique (AFS impact, AWETA, G&P, Nootdorp, The Netherlands). In this method only two parameters are needed to obtain the firmness index (FI): the resonant frequency of the first elliptical mode and the mass of the fruit [23,24]. In experiment 2 each sample was measured at two opposite sites and as a result the mean value of FI for each fruit was obtained.

Moreover, in experiment 2, apple firmness was also measured destructively using a universal testing machine Lloyd LRX (Lloyd Instruments Ltd, Hampshire, UK) with a 500 N load cell. Ten apples were punctured by a cylindrical Magness-Taylor probe (11.1 mm diameter) at a speed of 20 mm min^−1^. Before the test, the apple skin was removed. Maximum force, needed to penetrate the flesh over a distance of 8 mm was read as the apple firmness (F) and expressed in N.

### 2.3. Soluble Solids Content

Soluble solids content (SSC) was determined using a digital refractometer (PAL-BX/RI, Atago Co. Ltd., Tokyo, Japan). Freshly squeezed juice was poured onto the prism. The results were obtained in Brix degrees. Measurements were carried out for five apples in four replicates.

### 2.4. Dry Matter Content

Samples of five apples (approximately 20 g) were cut into small pieces and dried (SUP-30W, Wamed, Poland) at 105 °C to constant mass. Dry matter content (DM) was calculated as DM = (m_2_/m_1_) × 100, where m_1_ was the mass of the fresh sample and m_2_—mass of the dried sample.

### 2.5. Statistical Analysis

Statistical analysis was performed using Statistica 8.0 (StatSoft, Inc., Tulsa, OK, USA). In the experiment 1 storage effect was tested by one-way ANOVA to show differences between measured biospeckle activity during apple fungal disease development. *Post-hoc* analysis were performed using the HSD Tukey test. In the experiment 2 basic statistics (mean values and standard deviations) were calculated for quality attributes and biospeckle activity measured in the shelf life simulations. Moreover two-way ANOVA was used for testing series*day effect. Pearson’s correlation coefficients between mean BA values and other mean quality attributes were also determined. The effects were tested using *F-value* and the significance level was evaluated at *p* < 0.05.

## 3. Results

### 3.1. Infection Development in Experiment 1

**Figure 2.**
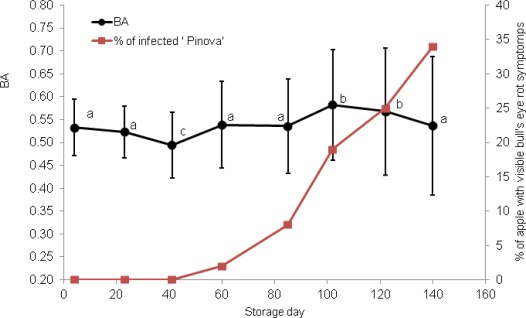
Biospeckle activity BA (black line) and percentage of infected fruit (red line) during development of bull’s eye rot of ‘Pinova’ apples. Bars indicate standard deviation; different letters denote significant differences (at α = 0.05) between means.

**Figure 3.**
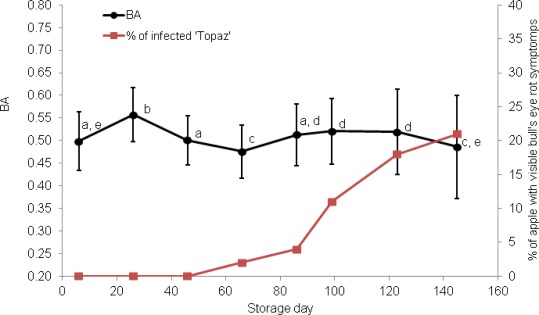
Biospeckle activity BA (black line) and percentage of infected fruit (red line) during development of bull’s eye rot of ‘Topaz’ apples. Bars indicate standard deviation; different letters denote significant differences (at α = 0.05) between means.

After about two months of storage, the first hardly visible spots appeared on the skin of two apples of both cultivars (Figures 2 and 3).

The circular and light-brown areas constantly increased in size and darkened with time. As a result, 34% of ‘Pinova’ and 21% of ’Topaz’ apples were severely diseased after ∼140 d. Symptoms of infections were always more pronounced in ‘Pinova’ apples (Figure 2). Bull’s eye rots in this case were mostly observed close to the equator of apples, whereas in ‘Topaz’, the spots usually appeared close to the stem or calyx end.

**Figure 4.**
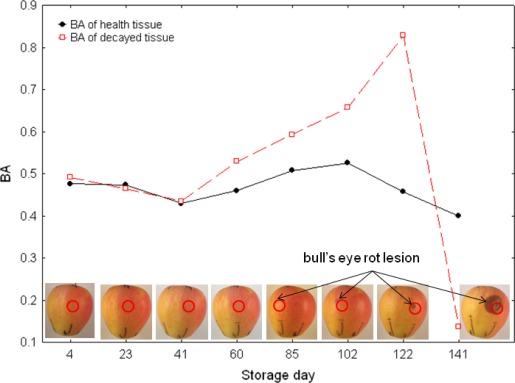
Biospeckle activity BA of one selected ‘Pinova’ apples for healthy and decaying tissue during storage. Photographic documentation of apple bull’s eye rot development is also given. Lesion area is marked by black arrow and red circle presents laser illumination point.

Each mean BA value, presented in Figures 2 and 3, was calculated from 600 measurements (100 apples × 6 places on the equator). Standard deviations of BA are relatively large however due to number of repetitions observed changes were often significant and allowed making conclusions about trends. Overall effect of storage showed significant change of biospeckle activity (F-value > 38, *p* < 0.05). With one exception, BA change trends were similar for apples of both cultivars. On day 26 of storage, however, BA of ‘Topaz’ apples occasionally increased (Figure 3). Despite this, BA of healthy apples seemed to decrease (‘Pinova’) or, at least, remained constant (‘Topaz’) during the initial 40 d of storage. In the next stage, (∼40–100 storage days) BA significantly increased as the *post-hoc* analysis showed. In this period small, light–brown, circular spots appeared. Prolonged storage (∼100–140 d) caused pronounced visible symptoms and death of the tissue. This is accompanied by significant decrease of biospeckle activity (Figure 4).

Due to the experimental set up, the locations of BA measurements were preliminary fixed and were monitored in the same places. Since bull’s eye rot development was not controlled, as a result, direct BA measurements of bull’s eye rot spots exactly in the same place were very rare. Comparison of biospeckle activity changes for infected and health place on the same apple in a specific case where rotting appeared exactly in monitoring area (Figure 4) confirmed results in Figures 2 and 3. BA decreased during the 40 d of cold storage. Then in both healthy and infected places biospeckle activity increased but for the decaying place it happened faster. The first bull’s eye rot hardly visible symptoms occurred at 85th day of NA storage in this case. It suggests that maybe the increase of BA at 60th day, when the change 40–60 d was already significant, would be used for prediction of infection. Finally, BA decreased and especially infected part showed very low activity.

### 3.2. Biospeckle Activity after Various Storage Methods (Experiment 2)

Decrease in BA during shelf life was observed for 1NA series of both tested cultivars. In ‘Pinova’ apples, biospeckle activity changed slightly during 2NA shelf life simulation and increased during 3NA (Table 1). The highest value of biospeckle activity was obtained in general for 4CA series but also a decrease of BA was noted. After 1-MCP treatment (5MCP) biospeckle activity also decreased. BA of ‘Topaz’ decreased in the case of 1NA and 2NA shelf-life experiments (Table 2). For 3NA biospeckle activity increased from 0.48 (1st day) to 0.59 (10th day). During storage under 4CA and 5MCP conditions, no clear trend in changes of BA could be detected. Firmness gradually decreased in the case of 1NA, 2NA, 4CA and 5MCP, both in ‘Pinova’ (Table 1) and in ‘Topaz’ apples (Table 2). The lowest values of IF and F and the least pronounced shelf life effect was obtained in the 3NA experimental stage. Dry matter content DMC and total soluble solids content SSC generally increased during apple storage. The highest values of DMC were obtained at day 10 in the 5MCP series (18.60% for ‘Pinova’ and 18.03% for ‘Topaz’). 2NA program was characterized by the highest mean SSC, and it fluctuated from 14.2 to 15.7° Brix for cv. ‘Pinova’ and 12.7–14.1° Brix for cv. ‘Topaz’. In turn the lowest mean values of SSC was obtained for 4CA series in both cases.

Two-way ANOVA (Table 3) showed that combined effects of storage method and shelf life were significant (*p* < 0.05) for measured parameters with the exception of DMC for ‘Pinova’ (*p* = 0.19). Table 4 presents summarized Pearsons’ correlation coefficients (R) calculated among mean values of variables analyzed in this experiment. Strong correlation between BA and FI was observed for ‘Topaz’ (*R* = 0.84, *p* < 0.05), whereas in other cases *R*-values were weak but still significant at the level of *p* < 0.05. For ‘Pinova’ apples, correlations between BA and quality attributes were not significant (*p* > 0.05) with the exception of SSC, for which *R* = −0.40 (*p* < 0.05).

## 4. Discussion

It is believed that biospeckle fluctuations result from the elastic light scattering on moving particles such as cellular organelles. Any disturbance of this movement would be detected as a BA change. Thus, biospeckle activity fluctuation, presented in Figures 2 and 3, may be related to pathological changes occurring in apple tissues during the development of fungal infection. In the first period, before visible symptoms appeared, BA decreased, probably due to starch granule degradation [18]. The decrease of biospeckle activity during storage was previously reported by other authors [7,8,11,14,16,17].

According to the literature data [25,26] fungal attack results in elevated level of ethylene concentration and increase in transpiration and this could be a reason for observed increase in biospeckle activity between the ∼40th and ∼100th day.

During infection, a number of biologically active substances, like enzymes, toxins and growth regulators, are released by the fungal pathogens, which may affect the structural integrity of the host cells or their physiological process. Production of pectolytic enzymes causes pectin degradation and leads to plant tissue maceration, *i.e.*, softening, loss of coherence and separation of individual cells, which eventually die [25,27,28]. Death of the apple tissue was responsible for decrease in biospeckle activity (Figure 4) during prolonged storage (∼100–140 d).

During apple ripening many biochemical reactions take place that could be responsible for variable biospeckle. Chlorophyll degradation causes a BA increase due to a decrease of light absorption and deeper light penetration [19], whereas starch degradation has an opposite effect: BA increases due to decreases in the number of particles acting as light scattering centers [18]. These two processes usually occur during fruit ripening. In previous studies apparent biospeckle activity decreased just after harvest in shelf life experiments [17]. Similar results were obtained in present experiment, suggesting that starch hydrolysis plays a more important role than chlorophyll degradation for biospeckle activity. NA, CA and 1-MCP treatments are followed by a decrease in BA during shelf life. Table 4 showed that soluble solids content also affects biospeckle activity: higher SSC was reflected as lower BA. However, at present it is difficult to interpret the reasons behind this relationship.

## 5. Conclusions

This work revealed that biospeckle reflects biological activity occurring inside apples and on apple skins during bull’s eye rot development. An increase of biospeckle activity was observed when disease symptoms were hardly visible. Senescence resulted in a decline of biospeckle activity due to reduction of life processes within tissue. This showed that this method would be used in a future for non-destructive monitoring of pathogen infection development. This experiment showed that a limit of detection would be at least comparable to that of visual inspection. However to estimate this limit precisely further study is needed in model experiments, with artificial inoculation, where biospeckle activity will be evaluated exactly in the place of infection.

This study also showed that, irrespective of storage conditions, biospeckle activity decreased during shelf life. In the case of ‘Topaz’ BA correlated significantly (*p* < 0.05) with quality attributes (firmness, firmness index, soluble solids content, dry matter content), whereas for ‘Pinova’ a significant correlation was obtained only between BA and SSC. It can be concluded that there is a chance to monitor postharvest quality of apples, in relation to biochemical changes, by this non-destructive method.

## Figures and Tables

**Table 1.** Mean values of biospeckle activity (BA) and quality attributes for ‘Pinova’ (F–firmness, FI–firmness index, SSC–soluble solids content, DMC–dry matter content) of apple during shelf-life. SD–standard deviation, the same letters a–e mean no significant difference at the level of α = 0.05 between superscripted values.

**Series**	**Shelf life (days)**	**BA ± SD**	**F (N) ± SD**	**FI ± SD**	**SSC (°Brix) ± SD**	**DMC (%) ± SD**
1 NA	1	0.65 ± 0.06 ^a^	82.02 ± 4.44 ^a^	28.2 ± 2.5 ^a^	14.5 ± 0.8 ^ab^	13.85 ± 0.96 ^a^
	3	0.60 ± 0.04 ^b^	80.04 ± 3.23 ^a^	22.9 ± 2.4 ^b^	14.3 ± 0.7 ^abc^	14.82 ± 0.41 ^a^
	5	0.55 ± 0.06 ^c^	77.57 ± 6.38 ^a^	17.8 ± 1.9 ^c^	13.9 ± 0.5 ^c^	14.56 ± 1.54 ^a^
	7	0.53 ± 0.04 ^c^	77.79 ± 4.42 ^a^	15.7 ± 3.7 ^c^	14.1 ± 0.6 ^bc^	14.87 ± 1.55 ^a^
	10	0.55 ± 0.07 ^d^	77.27 ± 2.98 ^a^	11.0 ± 3.7 ^d^	14.7 ± 0.8 ^a^	14.78 ± 077 ^a^

2 NA	1	0.55 ± 0.05 ^ab^	56.66 ± 7.83 ^a^	15.3 ± 2.6 ^a^	14.2 ± 0.5 ^a^	14.28 ± 0.93 ^a^
	3	0.57 ± 0.05 ^b^	51.59 ± 6.41 ^a^	11.0 ± 3.1 ^b^	14.9 ± 0.6 ^ab^	14.41 ± 0.72 ^a^
	5	0.51 ± 0.04 ^a^	51.62 ± 4.64 ^a^	9.2 ± 2.0 ^c^	15.1 ± 1.3 ^bc^	14.95 ± 0.78 ^a^
	7	0.52 ± 0.07 ^a^	52.72 ± 7.37 ^a^	6.3 ± 2.0 ^d^	15.7 ± 0.8 ^c^	15.69 ± 1.00 ^a^
	10	0.58 ± 0.05 ^b^	52.15 ± 6.26 ^a^	5.1 ± 1.5 ^d^	15.2 ± 1.1 ^bc^	15.10 ± 0.94 ^a^

3 NA	1	0.62 ± 0.11 ^a^	35.28 ± 5.32 ^a^	4.9 ± 1.6 ^a^	14.5 ± 1.1 ^ab^	15.32 ± 1.14 ^a^
	3	0.65 ± 0.10 ^ab^	32.58 ± 6.85 ^a^	4.4 ± 0.4 ^ab^	13.8 ± 0.8 ^a^	15.04 ± 0.85 ^a^
	5	0.62 ± 0.08 ^a^	31.34 ± 4.25 ^a^	4.1 ± 0.4 ^b^	14.9 ± 0.8 ^b^	13.93 ± 0.85 ^a^
	7	0.65 ± 0.12 ^ab^	33.38 ± 3.80 ^a^	3.9 ± 0.3 ^b^	15.2 ± 0.9 ^b^	14.87 ± 1.39 ^a^
	10	0.69 ± 0.09 ^b^	31.30 ± 4.61 ^a^	4.0 ± 0.3 ^b^	14.7 ± 0.6 ^b^	14.84 ± 1.01 ^a^

4 CA	1	0.73 ± 0.04 ^a^	71.66 ± 6.78 ^a^	20.0 ± 2.9 ^a^	13.7 ± 0.6 ^a^	15.34 ± 0.71 ^ab^
	3	0.66 ± 0.06 ^b^	74.18 ± 10.10 ^a^	16.4 ± 3.8 ^b^	14.7 ± 0.9 ^b^	14.31 ± 1.27 ^a^
	5	0.67 ± 0.05 ^b^	73.11 ± 7.35 ^a^	12.4 ± 2.9 ^c^	13.7 ± 1.1 ^a^	15.39 ± 1.09 ^ab^
	7	0.66 ± 0.07 ^b^	74.13 ± 3.19 ^a^	11.1 ± 2.9 ^c^	13.8 ± 0.8 ^a^	16.03 ± 0.56 ^b^
	10	0.69 ± 0.09 ^ab^	77.26 ± 8.27 ^a^	7.5 ± 1.7 ^d^	13.2 ± 1.1 ^a^	18.39 ± 0.64 ^b^

5 MCP	1	0.62 ± 0.09 ^ab^	85.72 ±10.41 ^ab^	19.2 ± 2.7 ^a^	14.1 ± 0.9 ^ab^	17.28 ± 1.26 ^a^
	3	0.65 ± 0.07 ^bc^	71.30 ± 19.74 ^a^	13.3 ± 3.0 ^b^	13.9 ± 1.0 ^a^	17.85 ± 0.75 ^a^
	5	0.67 ± 0.06 ^c^	88.20 ± 7.34 ^b^	12.1 ± 3.5 ^b^	15.1 ± 0.9 ^c^	17.29 ± 1.18 ^a^
	7	0.63 ±0.07 ^abc^	90.32 ± 10.20 ^b^	8.6 ± 2.6 ^c^	14.7 ± 0.4 ^bc^	18.02 ± 0.42 ^a^
	10	0.59 ± 0.08 ^a^	91.62 ± 10.11 ^b^	8.0 ± 1.6 ^c^	14.8 ± 1.0 ^bc^	18.60 ± 0.97 ^a^

**Table 2.** Mean values of biospeckle activity (BA) and quality attributes for ‘Topaz’ (F–firmness, FI–firmness index, SSC–soluble solids content, DMC–dry matter content) of apple during shelf-life. SD–standard deviation, the same letters a–e mean no significant difference at the level of α = 0.05 between superscripted values.

**Series**	**Shelf life (days)**	**BA ± SD**	**F (N) ± SD**	**FI ± SD**	**SSC (°Brix) ± SD**	**DMC (%) ± SD**
1 NA	1	0.64 ± 0.04 ^a^	85.98 ± 4.40 ^a^	25.6 ± 1.5 ^a^	12.7 ± 0.7 ^a^	12.92 ± 1.26 ^a^
	3	0.54 ± 0.08 ^bd^	75.48 ±18.86 ^ab^	22.1 ± 1.7 ^b^	13.4 ± 0.8 ^b^	12.77 ± 0.77 ^a^
	5	0.59 ± 0.05 ^c^	62.68 ±10.32 ^bc^	20.0 ± 1.7 ^c^	13.2 ± 0.6 ^ab^	13.90 ± 0.77 ^a^
	7	0.51 ± 0.06 ^d^	54.59 ± 6.05 ^cd^	15.8 ± 1.9 ^d^	12.7 ± 0.5 ^a^	13.10 ± 0.35 ^a^
	10	0.56 ± 0.05 ^bc^	47.57 ± 3.59 ^d^	12.4 ± 2.5 ^e^	13.6 ± 0.7 ^b^	12.84 ± 0.52 ^a^

2 NA	1	0.59 ± 0.06 ^a^	48.50 ± 2.72 ^a^	18.0 ± 1.7 ^a^	12.7 ± 1.0 ^a^	12.80 ± 0.23 ^a^
	3	0.55 ± 0.05 ^ab^	45.81 ± 3.88 ^a^	15.2 ± 3.1 ^b^	13.9 ± 1.0 ^b^	12.55 ± 0.69 ^a^
	5	0.57 ± 0.06 ^a^	45,95 ± 4.81 ^a^	13.6 ± 2.6 ^b^	13.6 ± 1.0 ^b^	13.45 ± 0.53 ^a^
	7	0.51 ± 0.06 ^b^	39.30 ± 2.36 ^b^	9.4 ± 1.9 ^c^	14.1 ± 0.9 ^b^	13.24 ± 0.79 ^a^
	10	0.57 ± 0.06 ^a^	38.32 ± 3.77 ^b^	7.9 ± 2.4 ^c^	13.6 ± 0.5 ^b^	13.71 ± 0.74 ^a^

3 NA	1	0.48 ± 0.05 ^ab^	35.32 ± 4.40 ^ab^	8.2 ± 2.6 ^ac^	12.6 ± 0.6 ^a^	12.39 ± 1.04 ^a^
	3	0.47 ± 0.05 ^a^	38.28 ± 3.87 ^a^	10.1 ± 2.7 ^ab^	13.8 ± 0.6 ^bc^	13.11 ± 0.41 ^a^
	5	0.53 ± 0.10 ^bc^	36.88 ± 5.03 ^a^	10.5 ± 3.1 ^b^	14.0 ± 0.7 ^b^	13.11 ± 0.79 ^a^
	7	0.57 ± 0.08 ^cd^	31.11 ± 3.45 ^bc^	7.8 ± 2.3 ^c^	13.3 ± 1.0 ^cd^	12.81 ± 0.86 ^a^
	10	0.59 ± 0.08 ^d^	29.73 ± 4.97 ^c^	7.8 ± 2.6 ^c^	13.0 ± 0.6 ^ad^	13.53 ± 0.31 ^a^

4 CA	1	0.66 ± 0.08 ^a^	52.87 ± 7.72 ^a^	21.1 ± 1.9 ^a^	12.1 ± 0.9 ^a^	14.82 ± 1.29 ^ab^
	3	0.60 ± 0.05 ^b^	51.55 ± 7.39 ^a^	20.3 ± 2.1 ^ab^	12.3 ± 0.4 ^ab^	13.68 ± 0.83 ^a^
	5	0.61 ± 0.05 ^b^	43.88 ± 6.24 ^ab^	19.8 ± 2.3 ^abc^	12.3 ± 0.5 ^ab^	13.84 ± 0.62 ^ab^
	7	0.59 ± 0.07 ^b^	44.34 ± 5.44 ^ab^	18.8 ± 3.1 ^bc^	12.6 ± 0.6 ^b^	14.81 ± 1.11 ^ab^
	10	0.63 ± 0.07 ^ab^	37.88 ± 9.27 ^b^	17.9 ± 3.3 ^c^	12 5 ± 0.6 ^ab^	15.82 ± 0.70 ^b^

5 MCP	1	0.59 ± 0.08 ^ac^	65.04 ± 8.69 ^ab^	22.8 ± 1.5 ^a^	13.1 ± 0.8 ^ab^	15.38 ± 0.52 ^a^
	3	0.64 ± 0.05 ^ab^	75.86 ± 12.94 ^a^	21.3 ± 1.7 ^b^	13.7 ± 0.5 ^a^	14.68 ± 0.58 ^a^
	5	0.66 ± 0.07 ^b^	62.22 ± 5.43 ^b^	20.9 ± 2.3 ^b^	13.0 ± 0.9 ^ab^	14.59 ± 0.45 ^a^
	7	0.58 ± 0.08 ^c^	54.70 ± 7.13 ^b^	19.1 ± 2.3 ^c^	13.0 ± 0.8 ^ab^	15.26 ± 1.20 ^a^
	10	0.59 ± 0.08 ^ac^	61.06 ± 15.32 ^b^	20.8 ± 1.8 ^b^	12.6 ± 1.0 ^b^	18.03 ± 0.81 ^b^

**Table 3.** *F*-value and *p*-values of two-way ANOVA for combined series*day effect for individual cultivars. BA means biospeckle activity, F–firmness, FI–firmness index, SSC–soluble solids content, DMC–dry matter content.

Cultivar	***BA***	***F(N)***	***FI***	***SSC***	***DMC***
Pinova	*F-*value = 6.59, *p* < 0.05	*F-*value = 2.79, *p* < 0.05	*F-*value = 27.05, *p* < 0.05	*F-*value = 6.9, *p* < 0.05	*F-*value = 1.34, *p* = 0.19
Topaz	*F-*value = 10.41, *p* < 0.05	*F-*value = 5.74, *p* < 0.05	*F-*value = 26.47, *p* < 0.05	*F-*value = 4.7, *p* < 0.05	*F-*value = 3.22, *p* < 0.05

**Table 4.** Pearson’s correlation coefficients (*R*) for the correlation between biospeckle activity (BA) and other quality attributes (F–firmness, FI–firmness index, SSC–soluble solids content, DMC–dry matter content), measured for ‘Pinova’ and ‘Topaz’ apples. Asterisks denote significance at *p* < 0.05.

	**BA ‘Pinova’**	**BA ’Topaz’**
**FI**	0.02	0.84 *
**F**	0.03	0.42 *
**SSC**	−0.40 *	−0.48 *
**DMC**	0.19	0.52 *
